# Depressive symptoms and problematic alcohol and other substance use in 1476 gay, bisexual, and other MSM at three research sites in Kenya

**DOI:** 10.1097/QAD.0000000000001847

**Published:** 2018-07-04

**Authors:** Christine Korhonen, Makobu Kimani, Elizabeth Wahome, Fredrick Otieno, Duncan Okall, Robert C. Bailey, Gary W. Harper, Robert R. Lorway, Monika Doshi, John Mathenge, Joshua Kimani, Eduard J. Sanders, Susan M. Graham

**Affiliations:** a University of Washington, Seattle, Washington, USA; b Kenya Medical Research Institute, Nairobi; c Nyanza Reproductive Health Society, Kisumu, Kenya; d University of Illinois at Chicago, Chicago, Illinois; e University of Michigan, Ann Arbor, Michigan, USA; f University of Manitoba; g SAATH, Winnipeg, Manitoba, Canada; h Health Options for Young Men on HIV/AIDS and STIs; i University of Nairobi, Nairobi, Kenya; j University of Oxford, Oxford, UK.

**Keywords:** cross-sectional studies, depressive disorder, HIV infections, homosexuality, Kenya, male, substance-related disorders

## Abstract

**Objective:**

Information on mental health and substance use challenges among gay, bisexual, and other MSM (GBMSM) is needed to focus resources on these issues and optimize services for HIV prevention and care. We determined factors associated with depressive symptoms and problematic alcohol and other substance use among GBMSM in Kenya.

**Methods:**

Self-identified GBMSM in three HIV research studies in Kenya provided information on depressive symptoms [Patient Health Questionnaire 9 (PHQ-9)], alcohol use [Alcohol Use Disorder Identification Test (AUDIT)], and other substance use [Drug Abuse Screening Test 6 (DAST-6)]. Associations were evaluated using mixed effects Poisson regression.

**Results:**

Of 1476 participants, 452 (31%) reported moderate-to-severe depressive symptoms (PHQ-9 ≥ 10), 637 (44%) hazardous alcohol use (AUDIT ≥ 8), and 749 (51%) problematic substance use (DAST-6 ≥ 1). Known HIV-positive status was not associated with these outcomes. Transactional sex was associated with hazardous alcohol use [adjusted prevalence ratio (aPR) 1.34, 95% confidence interval (CI) 1.12–1.60]. Childhood abuse and recent trauma were associated with moderate-to-severe depressive symptoms (aPR 1.43, 95% CI 1.10–1.86 and aPR 2.43, 95% CI 1.91–3.09, respectively), hazardous alcohol use (aPR 1.36, 95% CI 1.10–1.68 and aPR 1.60, 95% CI 1.33–1.93, respectively), and problematic substance use (aPR 1.32, 95% CI 1.09–1.60 and aPR 1.35, 95% CI 1.14–1.59, respectively).

**Conclusion:**

GBMSM in rights-constrained settings need culturally appropriate services for treatment and prevention of mental health and substance use disorders, in addition to human rights advocacy to prevent abuse. Mental health and substance use screening and treatment or referral should be an integral part of programs, including HIV prevention and treatment programs, providing services to GBMSM.

## Introduction

Male–male sexual behavior is illegal in several African countries, including Kenya [1], where gay, bisexual, and other MSM (GBMSM) are targets of discrimination, blackmail, and violence [2,3]. This hostility can lead to high levels of distress, contributing to depression, alcohol use, and other substance use [4–7]. The minority stress theory posits that gay and bisexual individuals experience stress due to societal stigma, and attributes poor mental health to this stress [8]. Stressors may be more acute among people with intersecting minority status, such as GBMSM living with HIV infection and those engaging in transactional sex [9]. In addition, GBMSM recruited into HIV prevention studies in sub-Saharan Africa report high levels of childhood abuse and more recent trauma [3–5], each of which may contribute to adverse psychosocial outcomes.

Depressed mood, alcohol use, and other substance use are important predictors of sexual risk behavior among GBMSM in sub-Saharan Africa [7,10,11]. Information on psychosocial and contextual factors leading to these conditions is needed to develop and target effective prevention strategies tailored to this population [11]. We hypothesized that living with HIV and engaging in transaction sex would be associated with increased depressive symptoms, alcohol use, and other substance use among Kenyan GBMSM. Using cross-sectional data from three Kenyan research sites, the current study examines two questions. First, how prevalent are depressive symptoms, alcohol use, and substance use in a diverse population of GBMSM? Second, to what extent are HIV status, transactional sex, childhood abuse, and recent victimization associated with these three conditions?

## Methods

### Study sites

This is a secondary analysis of data abstracted from three studies of self-identified GBMSM conducted in Nairobi, coastal Kenya, and Kisumu by members of the GBMSM Health Research Consortium, a collaboration focused on improving HIV prevention and care services for GBMSM in Kenya. The geographic areas covered represent three distinct regions containing the majority of Kenya's multiethnic population. Studies in Nairobi and coastal Kenya targeted recruitment toward GBMSM engaged in transactional sex.

#### Nairobi

Participants were recruited for a cross-sectional mixed-methods study sponsored by the Canadian Institutes of Health Research to understand vulnerability to HIV in this population. Recruitment took place between January and May 2016 at two clinics providing tailored services to GBMSM.

#### Coastal Kenya

Participants were enrolled in cohorts at the Kenya Medical Research Institute-Wellcome Trust Research Programme in Mtwapa [4,12,13]. Sociodemographic data were collected at cohort enrollment. Mental health data were collected at enrollment or follow-up visits between December 2015 and October 2016.

#### Kisumu

Participants were recruited for a find, test, link, and retain in care study called ‘*Anza Mapema*’ (Kiswahili for ‘start early’), sponsored by the US Centers for Disease Control and Prevention. HIV-positive GBMSM already linked to care were excluded. Baseline mental health data were collected at enrollment visits between August 2015 and September 2016.

### Data collection

Data were collected by audio computer-assisted self-interview (ACASI) or computer-assisted personal interview (CAPI) in English, Kiswahili, or Dholuo. A version of the Patient Health Questionnaire 9 (PHQ-9) depression module validated in Kiswahili was used at all sites [14]. Other questions were translated from English to Kiswahili or Dholuo by staff at each site, with back-translation to ensure retained meaning. Questions considered unclear or difficult to translate were edited according to each site's local review process.

Trained interviewers explained procedures, answered questions, and assisted with technical problems. After completing the ACASI/CAPI, participants debriefed with a counselor and were provided referrals for mental health services as needed. HIV counseling and testing was then conducted according to Kenyan guidelines [15].

### Measures

#### Outcomes

##### Depressive symptoms

The PHQ-9, with nine items rated on a 4-point Likert scale, was used to assess depressive symptoms (Supplemental Table 1) [16]. The standard PHQ-9 responses of ‘Not at all’, ‘Several days’, ‘More than half the days’, and ‘Nearly every day’ were used in Kisumu, whereas the Nairobi and coastal Kenya sites used a revised response set: ‘Not at all’, ‘A few days’, ‘Several days’, and ‘Nearly all the days’. Responses were summed for a total score ranging from 0 to 27. Based on PHQ-9 scoring guidelines, a score of 10 or above defined moderate-to-severe depressive symptoms [16].

##### Alcohol use

The Alcohol Use Disorder Identification Test (AUDIT) rates 10 items on a 5-point scale (Supplemental Table 2) [17]. In Nairobi and coastal Kenya, if a participant answered ‘Never’ to the first item, on the frequency of drinking, the final two items were asked (refer to Supplemental Table 2). In Kisumu, no skip pattern was used. In Nairobi and coastal Kenya, the responses ‘Never’, ‘A few days a year’, ‘Every month’, ‘Every week’, and ‘Every day’ were used for questions 3–8. In Kisumu, the responses for these items were modified: ‘Never’, ‘Monthly or less’, ‘2–4 times a month’, ‘2–3 times a week’, and ‘4 or more times a week’. Responses were summed for a total score ranging from 0 to 40. A score of 8 or above (Zones II -IV) defined hazardous alcohol use [17].

##### Other substance use

The Drug Abuse Screening Test 10 (DAST-10) was used in Nairobi and coastal Kenya to measure problematic use of nonprescription substances other than alcohol or tobacco [18]. In Kisumu, a shortened instrument omitted four DAST-10 items (Supplemental Table 3). In Kisumu, a skip pattern was employed so that participants reporting no nonmedical substance use were asked no further items. For this analysis, only the six items (DAST-6) asked at all sites were used. A positive response on any item defined problematic substance use.

#### Primary exposures

##### HIV status

Self-reported HIV status was compared with HIV test results and separated into three categories: known HIV-positive (i.e. self-reported positive/tested positive), newly diagnosed (i.e. self-reported negative or unknown/tested positive), and HIV-negative (i.e. tested negative).

##### Transactional sex

Engagement in transactional sex was dichotomized to any engagement vs. none. Questions about transactional sex varied across sites (Supplemental Table 4).

#### Additional exposures

##### Childhood abuse

Childhood abuse was measured using the four-item Childhood Experience of Care and Abuse scale (Supplemental Table 5) [19]. A positive response to any item was classified as childhood abuse.

##### Recent trauma

Recent trauma was assessed using the four-item USAID Health Policy Initiative MSM Trauma Screening Tool (Supplemental Table 6) [20]. In Nairobi and coastal Kenya, questions asked about trauma in the past year. In Kisumu, questions asked about trauma in the past 3 months. A positive response to any item was classified as recent trauma.

#### Confounders

##### Sociodemographics

All sites asked about age, religion, education, and marital status.

### Data analysis

De-identified data from each site were standardized and merged into a unified dataset. For categorical variables, number and percentage were calculated and Pearson's Chi-square test performed for comparisons across HIV status groups. For continuous variables, median and interquartile range (IQR) were calculated and the Kruskal–Wallis test used to compare across HIV status groups. Spearman's rank-order correlation between outcomes was calculated. ‘Don’t know’, ‘refused’ and missing responses were relatively frequent (>10%) for engagement in transactional sex, childhood abuse, and recent trauma. Because participants may have refused or skipped questions for a reason, these responses were coded as a separate category, and data were not imputed.

Mixed effects Poisson regression with a random effect for site was used to produce prevalence ratios in both univariable and multivariable analyses. HIV status, engagement in transactional sex, childhood abuse, and recent trauma were included *a priori* in all multivariable analyses. Age, marital status, education, and religion were included in multivariable analyses when associated with the outcome of interest at *P* values less than 0.20. *P* values for categorical variables were calculated using Wald tests. Data were analyzed using Stata version 14 (StataCorp, College Station, Texas, USA).

### Ethics statement

All participants provided written informed consent. Research protocols were approved by the Kenyatta National Hospital and University of Manitoba (Nairobi); Maseno University, University of Illinois at Chicago, and University of Washington (Kisumu); and Kenya Medical Research Institute and University of Washington (coastal Kenya). All sites formally agreed to share data.

## Results

### Study population

Results were available for 1476 participants: 537 (37%) from Nairobi, 241 (16%) from coastal Kenya, and 698 (47%) from Kisumu (Table 1). Median age was 25 years (IQR 22–29). A majority (932, 63%) reported engagement in transactional sex, as expected due to recruitment targeting GBMSM engaging in transactional sex at two sites. Two hundred and eighty-one participants (19%) reported living with HIV, of whom 264 (94%) tested positive (known HIV-positive) and 17 (6%) tested negative, contrary to their self-reported status. Among 1195 participants who self-reported as HIV-negative or status unknown, 128 (11%) tested HIV-positive (newly diagnosed HIV-positive). The remaining 1085 participants were HIV-negative. Across HIV status categories, hazardous alcohol use was less common among participants with known HIV-positive status (*P* = 0.001). HIV-negative participants had a higher frequency of reported childhood abuse (*P* = 0.01).

### Depressive symptoms, alcohol use, and other substance use

Median PHQ-9 score was 7 (IQR 3–11), and 452 participants (31%) had a PHQ-9 score of at least 10, compatible with moderate-to-severe depressive symptoms. Median AUDIT score was 6 (IQR 0–14), and 637 participants (44%) had an AUDIT score of at least 8, suggestive of hazardous alcohol use. Median DAST-6 score was 1 (IQR 0–4), and 749 participants (51%) had a DAST-6 score of at least 1, indicating problematic substance use. AUDIT and DAST-6 scores were weakly correlated with PHQ-9 scores (PHQ-9/AUDIT ρ = 0.27, *P* < 0.001; PHQ-9/DAST-6 ρ = 0.34, *P* < 0.001). AUDIT and DAST-6 scores were moderately correlated (ρ = 0.42, *P* < 0.001).

### Associations with moderate-to-severe depressive symptoms

In univariable analysis, childhood abuse and recent trauma were associated with moderate-to-severe depressive symptoms (Table 2). In multivariable analysis adjusted for religion and education, childhood abuse (adjusted prevalence ratio [aPR] 1.43, 95% confidence interval [CI] 1.10–1.86) and recent trauma (aPR 2.43, 95% CI 1.91–3.09) were associated with moderate-to-severe depressive symptoms.

### Associations with hazardous alcohol use

In univariable analysis, engagement in transactional sex, childhood abuse, and recent trauma were associated with hazard alcohol use (Table 3). In multivariable analysis, adjusted for age and education, transactional sex (aPR 1.34, 95% CI 1.12–1.60), childhood abuse (aPR 1.36, 95% CI 1.10–1.68), and recent trauma (aPR 1.60, 95% CI 1.33–1.93), were associated with hazardous alcohol use.

### Associations with problematic substance use

In univariable analysis, childhood abuse and recent trauma were associated with problematic substance use (Table 4). In multivariable analysis adjusted for religion, childhood abuse (aPR 1.32, 95% CI 1.09–1.60) and recent trauma (aPR 1.35, 95% CI 1.14–1.59) were associated with problematic substance use.

## Discussion

Given the multiple stressors faced by GBMSM across sub-Saharan Africa, a better understanding of factors influencing their mental health and substance use is needed. In a large, diverse population of GBMSM from three research sites in Kenya, we found that 31% reported moderate-to-severe depressive symptoms, 44% reported hazardous alcohol use, and 51% reported problematic substance use. Surprisingly, known HIV-positive status was not associated with any of these outcomes. Transactional sex was associated only with hazardous alcohol use. In contrast, both childhood abuse and recent trauma were associated with each of the outcomes studied, highlighting the importance of structural interventions to protect the rights of GBMSM.

The prevalence of moderate-to-severe depressive symptoms we found (31%) is higher than the 4.4% prevalence of major depressive disorder or dysthymia found in the general population of Kenyan males [21], but similar to the 34% prevalence of moderate-to-severe depressive symptoms among HIV-positive Kenyan men and women [22]. In addition, the 44% prevalence of hazardous alcohol use is higher than the 5.8% prevalence reported for men in the general Kenyan population [23]. We could find no comparable data on other substance use disorders in Kenyan men.

Transactional sex was associated with hazardous alcohol use, but not with depressive symptoms or problematic substance use. In qualitative research, men engaging in transactional sex in Kenya have reported drinking to excess as part of their work, which often involves meeting clients in bars [24]. Given the health effects of substance use, including increased risk of HIV acquisition, the high levels of both alcohol and other substance use we found suggest that interventions to address these problems and underlying factors are sorely needed [11].

Over 70% of study participants reported physical or sexual abuse in childhood. Similar to our findings, the 2010 Kenya Violence Against Children Study found that 18% of males reported sexual violence and 73% reported physical violence prior to age 18 [25]. We found that both childhood abuse and recent adult trauma were associated with moderate-to-severe depressive symptoms, hazardous alcohol use, and problematic substance use. These problems have profound and wide-ranging effects, and abuse counseling should be integral to GBMSM-focused services in Kenya and other rights-constrained settings [26].

The WHO and Kenya's National AIDS Control Council have called for reducing stigma and discrimination against GBMSM to improve health outcomes [27,28]. The illegality of same-sex behavior, which provides a license to harass and discriminate, is unacceptable. Although a case has been filed in the Kenyan high court by the National Gay and Lesbian Human Rights Commission contesting these laws [29], progress is slow. Although Kenya's Ministry of Health supports programs for GBMSM [30], GBMSM still face hostility and discrimination in many healthcare settings [24]. Sensitivity training has been shown to reduce homoprejudice in Kenyan healthcare workers [31] and should be scaled up. Interventions to improve resilience and coping strategies have reduced internalized homophobia among US GBMSM, but have not been assessed among African GBMSM.

The current study has several limitations. First, ACASI/CAPI differences across sites likely increased variability. We attempted to address this problem by including a random effect for site in analyses. Second, as the scales used to measure outcomes have not been validated among GBMSM in East Africa, applying standard cut points to identify moderate-to-severe depressive symptoms, hazardous alcohol use, and problematic substance use may overestimate or underestimate prevalence. Validation of mental health measures in this population is needed. Third, we did not assess symptoms of anxiety or posttraumatic stress, which may overlap with depressive symptoms assessed in the PHQ-9. We also did not assess resilience or other factors mitigating mental health problems. Fourth, participants reporting transactional sex may not identify as sex workers. We did not have information on self-identification as a sex worker at all sites, so could not analyze this predictor. Fifth, Kenyan GBMSM are highly mobile, and it is possible that a small number participated at more than one site. Fingerprint scans are used at two sites, but double enrolments could not be checked due to software incompatibility. Sixth, participants were recruited using snowball sampling and peer recruitment, biasing the sample in favor of men known to be GBMSM. Therefore, this population may not be representative of all Kenyan GBMSM. Finally, this is a cross-sectional study, and therefore, associations cannot be interpreted as causal.

In conclusion, GBMSM in rights-constrained settings need culturally appropriate mental health and substance use prevention and treatment services, in addition to advocacy and structural interventions to protect human rights. Mental health and substance use screening and treatment or referral should be an integral part of programs, including HIV prevention and treatment programs, providing services to GBMSM.

## Acknowledgements

We thank the staff at the *Anza Mapema* Study and the Nyanza Reproductive Health Society in Kisumu; the Sex Worker Outreach Programme (SWOP) and Health Options for Young Men on HIV/AIDS/STI (HOYMAS) in Nairobi; and the KEMRI-Wellcome Trust Research Programme (KWTRP) in Mtwapa and Kilifi, for their work on these studies. We also thank the study participants for their willingness to share their experiences in a research context.

All authors substantially contributed to the study's conception, design, and performance.

The current work was partially funded by IAVI with the generous support of USAID and other donors; a full list of IAVI donors is available at www.iavi.org. The contents of this manuscript are the responsibility of the authors and do not necessarily reflect the views of USAID or the US Government.

The current work was also supported through the sub-Saharan African Network for TB/HIV Research Excellence (SANTHE), a DELTAS Africa Initiative (grant no. DEL-15-006). The DELTAS Africa Initiative is an independent funding scheme of the African Academy of Sciences (AAS)'s Alliance for Accelerating Excellence in Science in Africa (AESA) and supported by the New Partnership for Africa's Development Planning and Coordinating Agency (NEPAD Agency) with funding from the Wellcome Trust (grant no. 107752/Z/15/Z) and the UK government.

The KWTRP at the Centre for Geographical Medicine Research-Kilifi is supported by core funding from the Wellcome Trust (no. 203077/Z/16/Z) and infrastructure funding from the University of Washington Center for AIDS Research (*AI027757)*. The Anza Mapema Study was supported through funding provided by the Centers for Disease Control and Prevention (U01GH000762) and by Evidence for HIV Prevention in Southern Africa (MM/EHPSA/NRHS/0515008). The South-to-South (S2S) study in Nairobi was supported through a grant from the Canadian Institutes of Health Research (CIHR operating grant no. HHP-131557).

The views expressed in this publication are those of the authors and do not necessarily reflect the views the US Government, UK government, AAS, NEPAD Agency, USAID, or the Wellcome Trust. This report was published with permission from the Director of KEMRI-Kilifi.

### Conflicts of interest

There are no conflicts of interest.

## Supplementary Material

Supplemental Digital Content

## Figures and Tables

**Table 1:** Characteristics of participants by HIV status (*n* = 1476).

Characteristic	Overall	Known HIV-positive	Newly diagnosed HIV-positive	HIV-negative	*P* value
	*n* (%) or median (IQR)	*n* (%) or median (IQR)	*n* (%) or median (IQR)	*n* (%) or median (IQR)	
Number	1476	264 (17.9)	128 (8.7)	1084 (73.4)	–
Transactional sex					0.09
Any	932 (63.1)	165 (62.5)	90 (70.3)	677 (62.5)	
None	504 (34.2)	87 (33.0)	34 (26.6)	383 (35.3)	
Nonresponse^a^	40 (2.7)	12 (4.6)	4 (3.1)	24 (2.2)	
Childhood abuse					0.01
Any	1042 (70.6)	175 (66.3)	84 (65.6)	783 (72.2)	
None	396 (26.8)	85 (32.2)	44 (34.4)	267 (24.6)	
Nonresponse^a^	38 (2.6)	4 (1.5)	0	34 (3.1)	
Recent trauma^b^					0.15
Any	756 (51.2)	138 (52.3)	58 (45.3)	560 (51.7)	
None	612 (41.5)	115 (43.6)	58 (45.3)	439 (40.5)	
Nonresponse^a^	108 (7.3)	11 (4.2)	12 (9.4)	85 (7.8)	
Age (years)	25 (22–29)	28 (24–32)	26 (23–31)	24 (21–28)	<0.001
Currently married to a woman^c^	135 (9.2)	24 (9.1)	10 (7.9)	101 (9.3)	0.86
Education^d^					0.17
Primary or less	513 (34.8)	105 (39.8)	44 (34.4)	364 (33.6)	
Completed secondary	505 (34.2)	79 (29.9)	38 (29.7)	388 (35.8)	
Some higher education	457 (31.0)	80 (30.3)	46 (35.9)	331 (30.6)	
Religion					0.08
Christian	1,058 (71.7)	200 (75.8)	99 (77.3)	759 (70.0)	
Muslim	186 (12.6)	32 (12.1)	17 (13.3)	137 (12.6)	
Other	112 (7.6)	14 (5.3)	9 (7.0)	89 (8.2)	
None	120 (8.1)	18 (6.8)	3 (2.3)	99 (9.1)	
Depressive symptoms (PHQ-9)^e^					0.76
Minimal (<5)	478 (32.4)	78 (29.6)	46 (35.9)	354 (32.7)	
Mild (5–9)	545 (37.0)	95 (36.0)	45 (35.2)	405 (37.4)	
Moderate (10–14)	258 (17.5)	51 (19.3)	20 (15.6)	187 (17.3)	
Moderately severe (15–19)	132 (9.0)	25 (9.5)	10 (7.8)	97 (9.0)	
Severe (≥20)	62 (4.2)	15 (5.7)	7 (5.5)	40 (3.7)	
Alcohol use risk level (AUDIT)^f^					0.001
Zone I (0–7)	828 (56.5)	166 (63.6)	76 (59.4)	586 (54.5)	
Zone II (8–15)	328 (22.4)	65 (24.9)	22 (17.2)	241 (22.4)	
Zone III (16–19)	97 (6.6)	14 (5.4)	8 (6.3)	75 (7.0)	
Zone IV (≥20)	212 (14.5)	16 (6.1)	22 (17.2)	174 (16.2)	
Any problematic substance use (DAST-6 ≥ 1)^g^	749 (50.8)	145 (54.9)	54 (42.2)	550 (51.0)	0.01

*Note:* Known HIV-positive was defined as self-reported positive and tested positive. Newly diagnosed HIV-positive was defined as self-reported negative and tested positive. HIV-negative was defined as tested negative. AUDIT, Alcohol Use Disorder Identification Test; DAST-6, Drug Abuse Screening Test 6; IQR, interquartile range; PHQ-9, Patient Health Questionnaire 9.

^a^Nonresponse included ‘don’t know’, ‘refused to answer’, and missing responses.

^b^Recent trauma was assessed over the past year in Nairobi and coastal Kenya and in the past 3 months in Kisumu.

^c^In Kenya, legal marriage is restricted to heterosexual relationships. Marital status was missing for four participants.

^d^Education was missing for one participant.

^e^PHQ-9 score was missing for one participant.

^f^AUDIT score was missing for 11 participants.

^g^DAST-6 score was missing for three participants.

**Table 2:** Factors associated with moderate-to-severe depressive symptoms.

	Univariable		Multivariable	
	Prevalence ratio (95% CI)	*P* value	Adjusted prevalence ratio^a^ (95% CI)	*P* value
*Primary exposures*				
HIV status^b^		0.64		0.37
Known positive	1.14 (0.86–1.49)		1.18 (0.94–1.49)	
Newly diagnosed positive	0.97 (0.69–1.37)		1.03 (0.73–1.46)	
Negative	ref		ref	
Transactional sex		0.06		0.51
Any	1.29 (1.05–1.59)		1.10 (0.89–1.35)	
Nonresponse^c^	1.28 (0.71–2.30)		1.33 (0.75–2.37)	
None	ref		ref	
*Secondary exposures*				
Childhood abuse		<0.001		0.02
Any	1.97 (1.53–2.54)		1.43 (1.10–1.86)	
Nonresponse^c^	2.08 (1.16–3.73)		1.62 (0.92–2.86)	
None	ref		ref	
Recent trauma		<0.001		<0.001
Any	2.76 (2.19–3.48)		2.43 (1.91–3.09)	
Nonresponse^c^	2.35 (1.58–3.50)		1.99 (1.35–2.93)	
None	ref		ref	
*Confounders*				
Age	1.01 (0.99–1.02)	0.25	–	
Currently married to a woman	0.92 (0.66–1.28)	0.62	–	
Education		0.11		0.30
Primary or less	ref		ref	
Completed secondary	0.76 (0.58–0.99)		0.83 (0.66–1.05)	
Some higher	0.85 (0.58–1.27)		0.94 (0.75–1.18)	
Religion		0.19		0.41
Christian	ref		ref	
Muslim	1.19 (0.91–1.56)		1.13 (0.86–1.49)	
Other	0.98 (0.68–1.42)		0.97 (0.67–1.39)	
None	1.37 (1.00–1.88)		1.27 (0.93–1.73)	

CI, confidence interval.

^a^Adjusted model includes engaged in transactional sex, childhood abuse, recent trauma, education, and religion.

^b^Known HIV-positive was defined as self-reported positive and tested positive. Newly diagnosed HIV-positive was defined as self-reported negative and tested positive. HIV-negative was defined as tested negative.

^c^Nonresponse included ‘don’t know’, ‘refused to answer’, and missing responses.

**Table 3:** Factors associated with hazardous alcohol use.

	Univariable		Multivariable	
	Prevalence ratio (95% CI)	*P* value	Adjusted prevalence ratio^a^ (95% CI)	*P* value
*Primary exposures*				
HIV status^b^		0.49		0.17
Known positive	0.87 (0.68–1.11)		0.80 (0.62–1.02)	
Newly diagnosed positive	0.90 (0.68–1.21)		0.87 (0.64–1.16)	
Negative	ref		ref	
Transactional sex		<0.001		0.01
Any	1.47 (1.23–1.75)		1.34 (1.12–1.60)	
Nonresponse^c^	1.01 (0.55–1.83)		0.96 (0.53–1.76)	
*Secondary exposures*				
Childhood abuse		<0.001		0.01
Any	1.63 (1.33–2.00)		1.36 (1.10–1.68)	
Nonresponse^c^	1.17 (0.64–2.16)		0.98 (0.53–1.80)	
None	ref		ref	
Recent trauma		<0.001		<0.001
Any	1.81 (1.52–2.15)		1.60 (1.33–1.93)	
Nonresponse^c^	1.53 (1.11–2.10)		1.47 (1.06–2.04)	
None	ref		ref	
*Confounders*				
Age	1.02 (1.01–1.03)	<0.001	1.02 (1.01–1.03)	0.01
Currently married to a woman	0.99 (0.75–1.29)	0.92	–	
Education		0.04		0.07
Primary or less	ref		ref	
Completed secondary	0.91 (0.75–1.11)		0.99 (0.81–1.20)	
Some higher	1.16 (0.96–1.41)		1.21 (1.00–1.47)	
Religion		0.94		
Christian	ref		–	
Muslim	1.06 (0.84–1.35)		–	
Other	1.07 (0.80–1.42)		–	
None	1.03 (0.77–1.39)		–	

CI, confidence interval.

^a^Adjusted model includes engaged in transactional sex, childhood abuse, recent trauma, age, and education.

^b^Known HIV-positive was defined as self-reported positive and tested positive. Newly diagnosed HIV-positive was defined as self-reported negative and tested positive. HIV-negative was defined as tested negative.

^c^Nonresponse included ‘don’t know’, ‘refused to answer’, and missing responses.

**Table 4:** Factors associated with problematic substance use.

	Univariable		Multivariable	
	Prevalence ratio (95% CI)	*P* value	Adjusted prevalence ratio^a^ (95% CI)	*P* value
*Primary exposures*				
HIV status^b^		0.38		0.50
Known positive	0.91 (0.74–1.10)		1.07 (0.78–1.48)	
Newly diagnosed positive	0.85 (0.64–1.13)		1.16 (0.87–1.53)	
Negative	ref		ref	
Transactional sex		0.20		0.58
Any	1.15 (0.98–1.35)		1.07 (0.91–1.26)	
Nonresponse^c^	1.21 (0.78–1.88)		1.21 (0.78–1.88)	
None	ref		ref	
*Secondary exposures*				
Childhood abuse		<0.001		0.01
Any	1.48 (1.24–1.78)		1.32 (1.09–1.60)	
Nonresponse^c^	1.55 (1.01–2.39)		1.39 (0.89–2.15)	
None	ref		ref	
Recent trauma		<0.001		0.002
Any	1.47 (1.26–1.72)		1.35 (1.14–1.59)	
Nonresponse^c^	1.27 (0.92–1.75)		1.18 (0.85–1.65)	
None	ref		ref	
*Confounders*				
Age	1.00 (0.98–1.01)	0.98	–	
Currently married to a woman	1.00 (0.77–1.29)	0.98	–	
Education		0.69		
Primary or less	ref		–	
Completed secondary	1.08 (0.91–1.28)		–	
Some higher	1.05 (0.87–1.27)		–	
Religion		0.02		0.03
Christian	ref		ref	
Muslim	1.36 (1.11–1.66)		1.35 (1.11–1.65)	
Other	0.97 (0.72–1.31)		0.95 (0.70–1.28)	
None	1.10 (0.84–1.43)		1.08 (0.82–1.39)	

CI, confidence interval.

^a^Adjusted model includes engaged in transactional sex, childhood abuse, recent trauma, and religion.

^b^Known HIV-positive was defined as self-reported positive and tested positive. Newly diagnosed HIV-positive was defined as self-reported negative and tested positive. HIV-negative was defined as tested negative.

^c^Nonresponse included ‘don’t know’, ‘refused to answer’, and missing responses.
