# Supplementary material for: Depressive symptoms and problematic alcohol and other substance use in 1476 gay, bisexual, and other MSM at three research sites in Kenya
Source: AIDS. 2018 Jul 4;32(11):1507–15. doi: 10.1097/QAD.0000000000001847 (PMC6150184; doi:10.1097/QAD.0000000000001847)
Supplement: Supplemental Digital Content [file aids-32-1507-s001.docx]

Supplemental Table 1. Patient Health Questionnaire-9 (PHQ-9) items by HIV status (n=1,475)

Each item was prefaced with “Over the last two weeks, how often have you...”

|  | **Overall**  n (%) | **Known  HIV-positive**  n (%) | **Unknown  HIV-positive**  n (%) | **HIV-negative**  n (%) |
| --- | --- | --- | --- | --- |
| Experienced little interest or pleasure in doing things | | | | |
| Not at all* | 431 (29.2) | 88 (33.3) | 43 (33.6) | 300 (27.7) |
| A few days | 675 (45.7) | 109 (41.3) | 52 (40.6) | 514 (47.4) |
| Several days | 189 (12.8) | 41 (15.5) | 9 (7.0) | 139 (12.8) |
| Nearly all the days | 180 (12.2) | 26 (9.9) | 24 (18.8) | 130 (12.0) |
| Felt down, depressed, or hopeless | | | | |
| Not at all | 489 (33.1) | 72 (27.3) | 42 (32.8) | 375 (34.6) |
| A few days | 614 (41.6) | 117 (44.3) | 56 (43.8) | 441 (40.7) |
| Several days | 231 (15.7) | 52 (19.7) | 15 (11.7) | 164 (15.1) |
| Nearly all the days | 142 (9.6) | 23 (8.7) | 15 (11.7) | 104 (9.6) |
| Had trouble falling or staying asleep, or sleeping too much | | | | |
| Not at all | 541 (36.7) | 84 (31.8) | 37 (28.9) | 420 (38.8) |
| A few days | 596 (40.4) | 117 (44.3) | 63 (49.2) | 416 (38.4) |
| Several days | 195 (13.2) | 46 (17.4) | 14 (10.9) | 135 (12.5) |
| Nearly all the days | 144 (9.8) | 17 (6.4) | 14 (10.9) | 113 (10.4) |
| Felt tired or had little energy | | | | |
| Not at all | 499 (33.8) | 66 (25.0) | 43 (33.6) | 390 (36.0) |
| A few days | 651 (44.1) | 136 (51.5) | 61 (47.7) | 454 (41.9) |
| Several days | 214 (14.5) | 35 (13.3) | 16 (12.5) | 163 (15.0) |
| Nearly all the days | 112 (7.6) | 27 (10.2) | 8 (6.3) | 77 (7.1) |
| Had poor appetite or have overeaten | | | | |
| Not at all | 646 (43.8) | 106 (40.2) | 56 (43.8) | 484 (44.7) |
| A few days | 562 (38.1) | 105 (39.8) | 53 (41.4) | 404 (37.3) |
| Several days | 172 (11.7) | 38 (14.4) | 12 (9.4) | 122 (11.3) |
| Nearly all the days | 96 (6.5) | 15 (5.7) | 7 (5.5) | 74 (6.8) |
| Felt bad about yourself—or that you are a failure or have let yourself or your family down | | | | |
| Not at all | 675 (45.7) | 108 (40.9) | 59 (46.1) | 508 (46.9) |
| A few days | 465 (31.5) | 83 (31.4) | 40 (31.3) | 342 (31.6) |
| Several days | 172 (11.7) | 41 (15.5) | 17 (13.3) | 114 (10.5) |
| Nearly all the days | 164 (11.1) | 32 (12.1) | 12 (9.4) | 120 (11.1) |
| Had trouble concentrating on things, such as reading the newspaper or watching television | | | | |
| Not at all | 694 (47.0) | 124 (47.0) | 61 (47.7) | 509 (47.0) |
| A few days | 480 (32.5) | 83 (31.4) | 40 (31.3) | 357 (32.9) |
| Several days | 154 (10.4) | 34 (12.9) | 13 (10.2) | 107 (9.9) |
| Nearly all the days | 148 (10.0) | 23 (8.7) | 14 (10.9) | 111 (10.2) |

| Experienced moving or speaking so slowly that other people could have noticed? Or the opposite—being so fidgety or restless that you have been moving around a lot more than usual | | | | |
| --- | --- | --- | --- | --- |
| Not at all | 798 (54.1) | 139 (52.7) | 67 (52.3) | 592 (54.6) |
| A few days | 451 (30.6) | 78 (29.6) | 41 (32.0) | 332 (30.6) |
| Several days | 140 (9.5) | 30 (11.4) | 13 (10.2) | 97 (9.0) |
| Nearly all the days | 87 (5.9) | 17 (6.4) | 7 (5.5) | 63 (5.8) |
| Thought that you would be better off dead or of hurting yourself in some way | | | | |
| Not at all | 978 (66.3) | 169 (64.0) | 81 (63.3) | 728 (67.2) |
| A few days | 301 (20.4) | 51 (19.3) | 32 (25.0) | 218 (20.1) |
| Several days | 122 (8.3) | 27 (10.2) | 8 (6.3) | 87 (8.0) |
| Nearly all the days | 75 (5.1) | 17 (6.4) | 7 (5.5) | 51 (4.7) |

*Responses in Kisumu: Not at all; Several days; More than half the days; Nearly every day.

Supplemental Table 2. Alcohol use disorders identification test (AUDIT) items by HIV status (n=1,476)

|  | **Overall**  n (%) | **Known  HIV-positive**  n (%) | **Unknown  HIV-positive**  n (%) | **HIV-negative**  n (%) |
| --- | --- | --- | --- | --- |
| How often do you have a drink containing alcohol | | | | |
| Never | 495 (33.5) | 85 (32.2) | 53 (41.4) | 357 (32.9) |
| Monthly or less | 256 (17.3) | 70 (26.5) | 20 (15.6) | 166 (15.3) |
| 2-4 times a month | 303 (20.5) | 58 (22.0) | 21 (16.4) | 224 (20.7) |
| 2-3 times a week | 264 (17.9) | 29 (11.0) | 17 (13.3) | 218 (20.1) |
| 4 or more times a week | 156 (10.6) | 22 (8.3) | 17 (13.3) | 117 (10.8) |
| Non-response | 2 (0.2) | 0 | 0 | 2 (0.2) |
| How many drinks containing alcohol do you have a on typical day when you are drinking | | | | |
| 1 to 2 | 453 (30.7) | 87 (33.0) | 31 (24.2) | 335 (30.9) |
| 3 to 4 | 339 (23.0) | 62 (23.5) | 25 (19.5) | 252 (23.3) |
| 5 to 6 | 137 (9.3) | 22 (8.3) | 9 (7.0) | 106 (9.8) |
| 7 to 9 | 25 (1.7) | 4 (1.5) | 3 (2.3) | 18 (1.7) |
| 10 or more | 25 (1.7) | 3 (1.1) | 7 (5.5) | 15 (1.4) |
| Non-response | 497 (33.7) | 86 (32.6) | 53 (41.4) | 358 (33.0) |
| How often do you have six or more drinks on one occasion | | | | |
| Never | 565 (38.3) | 90 (34.1) | 42 (32.8) | 433 (39.9) |
| A few days a year | 228 (15.5) | 47 (17.8) | 18 (14.1) | 163 (15.0) |
| Every month | 202 (13.7) | 21 (8.0) | 17 (13.3) | 164 (15.1) |
| Every week | 173 (11.7) | 25 (9.5) | 12 (9.4) | 136 (12.6) |
| Every day | 55 (3.7) | 4 (1.5) | 9 (7.0) | 42 (3.9) |
| Non-response | 253 (17.1) | 77 (29.2) | 30 (23.4) | 146 (13.5) |
| How often during the last year have you found that you were not able to stop drinking | | | | |
| Never | 686 (46.5) | 101 (38.3) | 54 (42.2) | 531 (49.0) |
| A few days a year | 195 (13.2) | 45 (17.1) | 14 (10.9) | 136 (12.6) |
| Every month | 137 (9.3) | 17 (6.4) | 10 (7.8) | 110 (10.2) |
| Every week | 108 (7.3) | 18 (6.8) | 10 (7.8) | 80 (7.4) |
| Every day | 95 (6.4) | 5 (1.9) | 10 (7.8) | 80 (7.4) |
| Non-response | 255 (17.3) | 78 (29.6) | 30 (23.4) | 147 (13.6) |
| How often during the last year have you failed to do what was expected of you because of drinking | | | | |
| Never | 753 (51.0) | 108 (40.9) | 57 (44.5) | 588 (54.2) |
| A few days a year | 231 (15.7) | 51 (19.3) | 13 (10.2) | 167 (15.4) |
| Every month | 123 (9.3) | 12 (4.6) | 17 (13.3) | 94 (9.7) |
| Every week | 74 (5.0) | 10 (3.8) | 6 (4.7) | 58 (5.4) |
| Every day | 42 (2.9) | 5 (1.9) | 5 (3.9) | 32 (3.0) |
| Non-response | 253 (17.1) | 78 (29.6) | 30 (23.4) | 145 (13.4) |
| How often during the last year have you needed a first drink in the morning | | | | |
| Never | 788 (53.4) | 135 (51.1) | 67 (52.3) | 586 (54.1) |
| A few days a year | 171 (11.6) | 33 (12.5) | 10 (7.8) | 128 (11.8) |
| Every month | 71 (4.8) | 6 (2.3) | 7 (5.5) | 58 (5.4) |
| Every week | 91 (6.2) | 7 (2.7) | 2 (1.6) | 82 (7.6) |
| Every day | 102 (6.9) | 6 (2.3) | 12 (9.4) | 84 (7.8) |
| Non-response | 253 (17.1) | 77 (29.2) | 30 (23.4) | 146 (13.5) |
| How often during the last year have you had a feeling of guilt after drinking | | | | |
| Never | 688 (46.6) | 96 (36.4) | 65 (50.8) | 527 (48.6) |
| A few days a year | 268 (18.2) | 63 (23.9) | 13 (10.2) | 192 (17.7) |
| Every month | 104 (7.1) | 13 (4.9) | 8 (6.3) | 83 (7.7) |
| Every week | 92 (6.2) | 5 (1.9) | 5 (3.9) | 82 (7.6) |
| Every day | 70 (4.7) | 10 (3.8) | 7 (5.5) | 53 (4.9) |
| Non-response | 254 (17.2) | 77 (29.2) | 30 (23.4) | 147 (13.6) |
| How often during the last year have you been unable to remember the night before | | | | |
| Never | 732 (49.6) | 109 (41.3) | 63 (49.2) | 560 (51.7) |
| A few days a year | 251 (17.0) | 60 (22.7) | 11 (8.6) | 180 (16.6) |
| Every month | 79 (5.4) | 7 (2.7) | 7 (5.5) | 65 (6.0) |
| Every week | 93 (6.3) | 7 (2.7) | 9 (7.0) | 77 (7.1) |
| Every day | 68 (4.6) | 4 (1.5) | 8 (6.3) | 56 (5.2) |
| Non-response | 253 (17.1) | 77 (29.2) | 30 (23.4) | 146 (13.5) |
| Have you or someone else been injured because of your drinking | | | | |
| No | 1091 (73.9) | 217 (82.2) | 93 (72.7) | 781 (72.1) |
| Yes, but not during the last year | 179 (12.1) | 23 (8.7) | 12 (9.4) | 144 (13.3) |
| Yes, during the last year | 205 (13.9) | 24 (9.1) | 23 (18.0) | 158 (14.6) |
| Non-response | 1 (0.1) | 0 | 0 | 1 (0.1) |
| Has someone been concerned about your drinking or suggested you cut down | | | | |
| No | 974 (66.0) | 176 (66.7) | 94 (73.4) | 704 (64.9) |
| Yes, but not during the last year | 198 (13.4) | 39 (14.8) | 10 (7.8) | 149 (13.8) |
| Yes, during the last year | 302 (20.5) | 48 (18.2) | 24 (18.8) | 230 (21.2) |
| Non-response | 2 (0.1) | 1 (0.4) | 0 | 1 (0.4) |

* Non-response included “don’t know,” “refused to answer,” missing responses, and participants who
 answered “never” to AUDIT item 1.

Supplemental Table 3. Drug Abuse Screening Test (DAST-6) items by HIV status (n=1,476)

|  | **Overall**  n (%) | **Known  HIV-positive**  n (%) | **Unknown  HIV-positive**  n (%) | **HIV-negative**  n (%) |
| --- | --- | --- | --- | --- |
| Have you used drugs other than those required for medical reasons | | | | |
| Yes | 506 (34.3) | 68 (25.8) | 40 (31.3) | 398 (36.3) |
| No | 970 (65.7) | 196 (74.2) | 88 (68.8) | 686 (63.3) |
| Non-response* | 0 | 0 | 0 | 0 |
| Have you used more than one drug at a time | | | | |
| Yes | 374 (25.3) | 79 (29.9) | 19 (14.8) | 276 (25.5) |
| No | 613 (41.5) | 159 (60.2) | 71 (55.5) | 383 (35.3) |
| Non-response | 489 (33.1) | 26 (9.9) | 38 (29.7) | 425 (39.2) |
| Have you always been able to stop using drugs when you want to | | | | |
| Yes | 517 (35.0) | 108 (40.9) | 32 (25.0) | 377 (34.8) |
| No | 466 (31.6) | 128 (48.5) | 58 (45.3) | 280 (25.8) |
| Non-response | 493 (33.4) | 28 (10.6) | 38 (29.7) | 427 (39.4) |
| Have you engaged in illegal activities in order to obtain drugs | | | | |
| Yes | 195 (13.2) | 29 (11.0) | 17 (13.3) | 149 (13.8) |
| No | 792 (53.7) | 208 (78.8) | 73 (57.0) | 511 (47.1) |
| Non-response | 489 (33.1) | 27 (10.2) | 38 (29.7) | 424 (39.1) |
| Have you ever experienced withdrawal symptoms when you stopped taking drugs | | | | |
| Yes | 267 (18.1) | 48 (18.2) | 19 (14.8) | 38 (29.7) |
| No | 718 (48.6) | 188 (71.2) | 71 (55.5) | 459 (48.6) |
| Non-response | 491 (33.3) | 28 (10.6) | 38 (29.7) | 425 (39.2) |
| Have you had medical problems as a result of your drug use? | | | | |
| Yes | 193 (13.1) | 36 (13.6) | 16 (13.6) | 141 (13.0) |
| No | 794 (53.8) | 201 (76.1) | 74 (57.8) | 519 (47.9) |
| Non-response | 489 (33.1) | 27 (10.2) | 38 (29.7) | 424 (39.1) |

* Non-response included “don’t know,” “refused to answer,” missing responses, and participants who
 answered “no” to DAST item 1 in Kisumu.

Supplemental Table 4. Questions used to assess engagement in transaction sex by site

| Nairobi: How long have you exchanged sex for money, food, clothes or other gifts? |
| --- |
| Kisumu: In the last three months, how often have you had sex with someone in order to get money, food or housing? |
| Coastal Kenya: In the last three months, have you been paid for sex with cash, living expenses, or goods? |

Supplemental Table 5. Childhood Experience of Care and Abuse (CECA) items by HIV status (n=1,476)

|  | **Overall**  n (%) | **Known  HIV-positive**  n (%) | **Unknown  HIV-positive**  n (%) | **HIV-negative**  n (%) |
| --- | --- | --- | --- | --- |
| When you were a child or teenager, were you ever hit repeatedly with an implement (such as a belt or stick) or punched, kicked, or burnt by someone in the household? | | | | |
| Yes | 882 (59.8) | 146 (55.3) | 67 (52.3) | 669 (61.7) |
| No | 559 (37.9) | 114 (43.2) | 61 (47.7) | 384 (35.4) |
| Non-response* | 35 (2.4) | 4 (1.5) | 0 | 31 (2.9) |
| When you were a child or teenager, did you ever have any unwanted sexual experiences? | | | | |
| Yes | 513 (34.8) | 90 (34.1) | 35 (27.3) | 388 (35.8) |
| No | 928 (62.9) | 170 (64.4) | 93 (72.7) | 665 (61.4) |
| Non-response | 35 (2.4) | 4 (1.5) | 0 | 31 (2.9) |
| Did anyone force you or persuade you to have sexual intercourse against your wishes before age 17? | | | | |
| Yes | 447 (30.3) | 83 (31.4) | 36 (28.1) | 328 (30.3) |
| No | 997 (67.6) | 177 (67.1) | 92 (71.9) | 728 (67.2) |
| Non-response | 32 (2.2) | 4 (1.5) | 0 | 28 (2.6) |
| Can you think of any upsetting sexual experiences before age 17 with a related adult or someone in authority (e.g. a teacher)? | | | | |
| Yes | 398 (27.0) | 81 (30.7) | 37 (28.9) | 250 (27.0) |
| No | 1046 (70.9) | 179 (67.8) | 91 (71.1) | 776 (71.6) |
| Non-response | 32 (2.2) | 4 (1.5) | 0 | 28 (2.6) |

*Non-response included “don’t know,” “refused to answer,” and missing responses

Supplemental Table 6. USAID Health Policy Initiative MSM Trauma Screening Tool items* by HIV status (n=1,476)

|  | **Overall**  n (%) | **Known HIV-positive**  n (%) | **Unknown HIV-positive**  n (%) | **HIV-negative**  n (%) |
| --- | --- | --- | --- | --- |
| Has anyone forced or coerced you to have sexual relations against your will? | | | | |
| Yes | 279 (18.9) | 62 (23.5) | 26 (20.3) | 191 (17.6) |
| No | 1147 (77.7) | 198 (75.0) | 94 (73.4) | 855 (78.9) |
| Non-response** | 50 (3.4) | 4 (1.5) | 8 (6.3) | 38 (3.5) |
| Has anyone slapped you, punched you, hit you, or caused you any other type of physical harm? | | | | |
| Yes | 350 (23.7) | 72 (27.3) | 30 (23.4) | 248 (22.9) |
| No | 1080 (73.2) | 186 (70.5) | 90 (70.3) | 804 (74.2) |
| Non-response | 46 (3.1) | 6 (2.3) | 8 (6.3) | 32 (3.0) |
| Has anyone insulted you, humiliated you, made you feel inadequate, or yelled at you? | | | | |
| Yes | 628 (42.6) | 105 (39.8) | 45 (35.2) | 478 (44.1) |
| No | 770 (52.2) | 152 (57.6) | 75 (58.6) | 543 (50.1) |
| Non-response | 78 (5.3) | 7 (2.7) | 8 (6.3) | 63 (5.8) |
| Has anyone made you feel threatened, fearful, or in danger? | | | | |
| Yes | 508 (34.4) | 104 (39.4) | 39 (30.5) | 365 (33.7) |
| No | 963 (65.2) | 156 (59.1) | 89 (69.5) | 718 (66.2) |
| Non-response | 5 (0.3) | 4 (1.5) | 0 | 1 (0.1) |

*Nairobi and Coastal Kenya questions prefaced with “In the past year,” Kisumu questions with “In the
 past three months.”

**Non-response included “don’t know,” “refused to answer,” and missing responses

Supplemental Table 7. Other substances used by site

| Substance | **Overall**  n=1476  n (%) | **Coastal Kenya**  n=241  n (%) | **Kisumu**  n=698  n (%) | **Nairobi**  n=537  n (%) |
| --- | --- | --- | --- | --- |
| Khat | 231 (15.7) | 63 (26.1) | 100 (14.5) | 68 (12.7) |
| Marijuana | 261 (17.7) | 45 (18.7) | 152 (22.1) | 64 (11.9) |
| Hashish | 13 (0.9) | 6 (2.5) | N/A | 7 (1.3) |
| Inhalants | 5 (0.3) | 1 (0.4) | N/A | 4 (0.7) |
| Heroin | 28 (1.9) | 7 (2.9) | 14 (2.0) | 7 (1.3) |
| Cocaine | 12 (0.8) | 2 (0.8) | 4 (0.6) | 6 (1.1) |
| Pain medications | 29 (2.0) | 13 (5.4) | N/A | 16 (3.0) |
| Sleeping medications | 33 (2.2) | 9 (3.7) | N/A | 24 (4.5) |
| Other | 15 (1.0) | 8 (3.3) | N/A | 7 (1.3) |
| Rohypnol | 3 (0.2) | N/A | 3 (0.4) | N/A |

N/A: Participants not asked about use of specific substance
